# Supplementary material for: A mirrorless spinwave resonator
Source: Sci Rep. 2015 Dec 10;5:17633. doi: 10.1038/srep17633 (PMC4674703; doi:10.1038/srep17633)
Supplement: Supplementary Information [file srep17633-s1.doc]

**A mirrorless spinwave resonator: Supplementary material**

**Olivier Pinel^+^, Jesse L. Everett^+^, Mahdi Hosseini, Geoff T. Campbell, Ben C. Buchler* and Ping Koy Lam***

Affiliation: Centre for Quantum Computation and Communication Technology, Department of Quantum Science, Research School of Physics and Engineering, The Australian National University, Canberra ACT 0200, Australia.

*corresponding authors: Ben.Buchler@anu.edu.au (BCB), Ping.Lam@anu.edu.au (PKL).

^+^ these authors contributed equally to this work

We briefly cover the experimental setup used to produce a gradient echo memory in a warm atomic vapour. We explain our data analysis, and provide numerical simulations based on the Maxwell-Bloch equations to illustrate how the atomic coherence builds up for resonant conditions.

**Methods**

The experimental setup is similar to that used in previous work [1-5]. Many of the experimental details can be found in our recent methods paper [6].

The laser source we use is a Ti:Sapphire laser (M-squared Solstis, pumped by Coherent Verdi) around 795 nm, detuned 2 GHz off the F=2 to F’=2 D1 transition. Part of the light is tapped off to form the control field. Another part is sent through a fiber EOM (EOSPACE), which modulates the light at 6.8 GHz – the ground state hyperfine splitting of Rb87.

A ring cavity is used to select the +6.8 GHz sideband of the modulated light. This sideband is used to produce both the probe and local oscillator (LO). Acousto-optic modulators (AOM, model MT80-A1-IR from A.A. Opto-electronic), in a double pass configuration, are used to further tune the frequencies and intensities of the probe, LO and control fields. The LO is detuned from the probe by ~2 MHz, and then recombined with the light coming out of the cell for heterodyne measurement. The probe 1/e^2^ diameter is 1.25 cm. The LO is larger to minimize noise due to beam pointing.

The probe and control are combined at a 90/10 beamsplitter, keeping most of the control field and discarding most of the probe. A cavity could also be used for lossless combining of the fields. Both fields are carefully circularly polarized using quarter- and half-waveplates and then sent through the cell. The control field is filtered after the rubidium 87 cell using an isotopically enhanced rubidium 85 cell, heated at 150° C – the control field is tuned to be resonant with the 5^2^S_1/2_ F=3-> 5^2^P_3/2_ F’=2 transition. The rubidium cell is heated with a non-magnetic resistive wire to a temperature of about 75°C. The heater is switched off during the experiment so the parasitic magnetic fields will not interfere with the experiment.

A constant axial magnetic field is applied to produce a certain level of ground state splitting. Longitudinal magnetic field gradients along the axis are applied using coils with increasing pitch – the frequency of probe at which the Raman transition will occur then depends on the position along the cell. Two coils with opposing gradients are used for storage and recall. The gradient coils are powered with low-voltage power supplies. They are switched using high-speed solid-state switches.

A typical experiment run consisted of sending a series of Gaussian pulses of the probe field through the Rubidium cell. The gradient was switched periodically at twice the pulse repetition rate. 15 pulses were sent, allowing the coherence to accumulate to equilibrium. The equilibrium coherence was measured by taking the final echo, which is proportional to the atomic coherence amplitude at that time. A new experimental run was performed every 60 ms.

**Decay and power accumulation measurements**

The control field frequency was set to maximize the accumulation of atomic coherence inside the cell. Pulse amplitudes were measured by their truncated heterodyne pulse areas, the limit between pulses determined from the minima of the heterodyne signal. The decay data was fitted with exponentials, and the error is the standard deviation of the residuals. The accumulation was normalized to the first echo obtained at each power level.

**Transmission spectra**

The control field was always scanned slowly enough that it could be considered constant over the duration of an experiment run. The amplitude of the last echo was measured from the heterodyne signal by taking the peak-to-peak value of the last echo. Full spectra were obtained by scanning the frequency of the control field by 800 kHz over a period of 200 s. The uncertainty of the measurements was computed from the local RMS noise of the trend-subtracted values.

The spectra of the central peak for linewidth and amplitude measurement were obtained by scanning 100 kHz over 10 s. Each measurement was repeated five times. Airy functions without the assumption of a high finesse cavity were used to fit these spectra.

The free spectral ranges for various pulse repetition rates were determined from the whole bandwidth data. The interval between peak values was fitted using a least-squares method. FSR error is approximated from phase noise by estimating how far in frequency the measured resonance might be from the actual resonance.

The equations that describe the gradient echo memory used in the experiment can be found in [5]. These equations can be solved numerically. We ran a simulation in one spatial dimension plus time, using XMDS [7]. We added an exponential decay term to the atomic coherence to approximate losses due to diffusion.


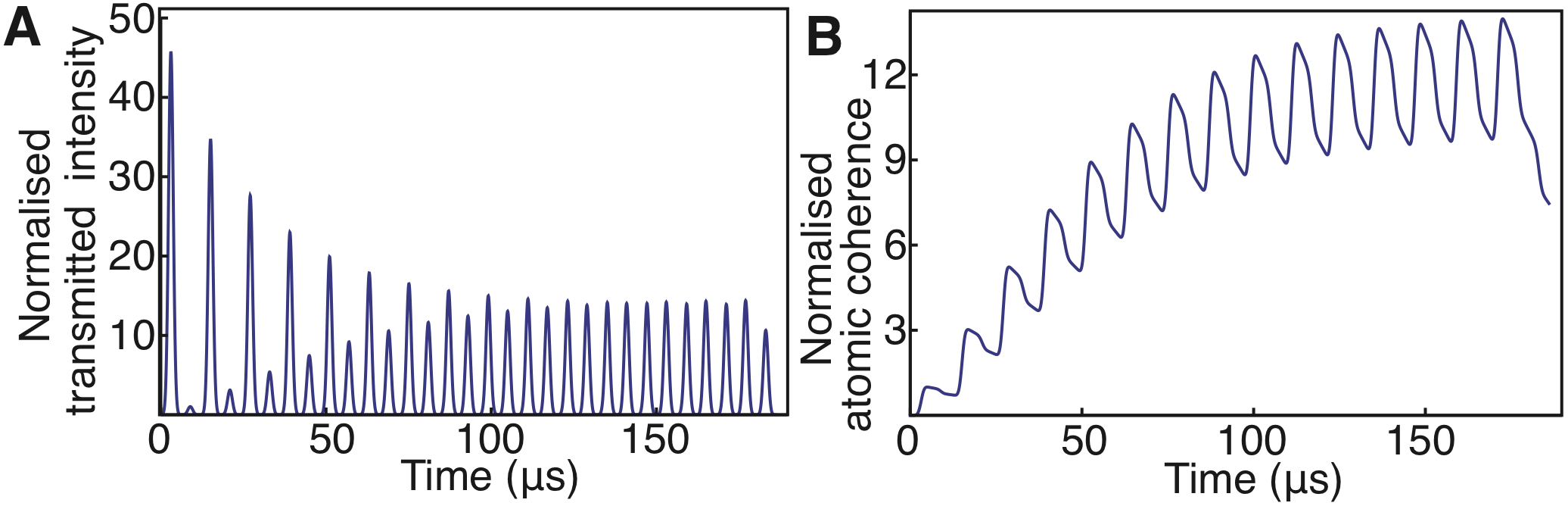


**Figure S1.** Simulation data. (A) Transmitted probe intensity for a series of pulses, scaled against the first echo. Leaked input pulses alternate with recalled pulses. (B) Atomic coherence magnitude for the same simulation. Both graphs are scaled against the first echo/storage in order to show the factor of 14 enhancement in the atomic coherence magnitude.

The results illustrate what occurs inside the memory as pulses are sent in. As the sent pulses are coupled in, the magnitude of the atomic coherence increases. As echo pulses are coupled out, the magnitude decreases. Decay due to other processes is apparent at other times. As the atomic coherence increases, the coupling in also increases due to the interference effect. This is apparent both as the larger increase in the atomic coherence at each coupling and as the decrease in the leakage of the sent pulse. Eventually, the other loss processes balance the increase, and equilibrium is reached.

**References**

1. M. Hosseini, B. M. Sparkes, G. Hetet, J. J. Longdell, P. K. Lam, and B. C. Buchler, Nature **461**, 241 (2009).
2. M. Hosseini, B. M. Sparkes, G. Campbell, P. K. Lam, and B. C. Buchler, Nat Comms **2**, 174 (2011).
3. M. Hosseini, G. Campbell, B. M. Sparkes, P. K. Lam, and B. C. Buchler, Nat Phys **7**, 794 (2011).
4. B. M. Sparkes, M. Hosseini, C. Cairns, D. Higginbottom, G. T. Campbell, P. K. Lam, and B. C. Buchler, Phys. Rev. X **2**, 021011 (2012).
5. M. Hosseini, B. M. Sparkes, G. T. Campbell, P. K. Lam, and B. C. Buchler, J Phys B-at Mol Opt **45**, 124004 (2012).
6. O. Pinel, M. Hosseini, B. M. Sparkes, J. L. Everett, D. Higginbottom, G. T. Campbell, P. K. Lam, and B. C. Buchler, Journal of Visualized Experiments e50552 (2013).
7. G. R. Dennis, J. J. Hope, and M. T. Johnsson, Computer Physics Communications **184**, 201 (2013).
